# Generative Language Models in Medical Education: From Advent to Entrustment

**DOI:** 10.2196/105347

**Published:** 2026-09-30

**Authors:** Shiv Patil, Mert Karabacak, Sotirios Bisdas, Konstantinos Margetis

**Affiliations:** 1 Sidney Kimmel Medical College Thomas Jefferson University Philadelphia, PA United States; 2 Department of Neurosurgery Loma Linda University Loma Linda, CA United States; 3 Department of Translational Neuroscience and Stroke Institute of Neurology University College London London, England United Kingdom; 4 Department of Neurosurgery Mount Sinai Hospital New York, NY United States

**Keywords:** generative artificial intelligence, medical education, large language models, assessment, AI literacy, governance, curriculum

## Abstract

Early discussions of generative language models in medical education emphasized their promise for simulation, digital patients, individualized feedback, learner assessment, health information dissemination, research support, and translation, while also warning about bias, privacy, academic integrity, misinformation, legal ambiguity, and unequal access. Since that first wave, generative AI has moved from novelty to routine exposure for learners, educators, researchers, and institutions. Medical education therefore needs a more mature framework than a catalog of opportunities and risks. This viewpoint argues that the next phase should be organized around educational entrustment: determining which functions can be delegated to AI systems, under what conditions, with what human supervision, and with what evidence of benefit. Building on recent proposals to apply entrustment to AI in health professions education, we operationalize the concept into a graduated, function-level model that specifies which educational functions may be delegated; at what stakes; and with what oversight, assessment, and governance. We classify use cases by educational stakes and AI autonomy and outline implications for assessment redesign, curriculum development, faculty capability, cognitive autonomy, equity, and institutional governance. The central challenge is whether medical schools can integrate these tools in ways that preserve clinical reasoning, professional identity, accountability, and fairness. The next generation of research should move beyond model performance on examinations and evaluate how AI changes learning, judgment, behavior, and patient care.

## Introduction: After the Advent

In 2023, generative language models entered medical education at an unprecedented pace. Our earlier viewpoint described a technology that could generate realistic patient scenarios, create digital patients, personalize feedback, assist with evaluation, improve access to health information, support research, and reduce language barriers [1]. It also warned that these benefits were inseparable from risks related to accuracy, bias, privacy, legal responsibility, academic dishonesty, transparency, and the digital divide. That framing remains useful because early enthusiasm often outpaced local policy and evidence. The field has since shifted from initial exploration toward operational decisions about which educational responsibilities can be entrusted to generative AI (GenAI), under what conditions, and with what forms of human oversight.

Several developments explain this shift. First, large language models (LLMs) have demonstrated rapidly improving performance on medical knowledge tasks, including licensing-style examinations and long-form medical question answering [2,3]. Second, generative systems have expanded from text to multimodal inputs and outputs. Models can increasingly combine language, images, audio, structured data, and conversational interfaces, expanding their relevance to radiology, pathology, physical examination teaching, clinical documentation, communication skills, and simulation [4,5]. Third, medical schools are moving from informal experimentation toward institutional policy, curriculum design, and assessment reform. Reviews and guides now converge on the need for AI literacy, educator development, ethical safeguards, and evaluation within authentic learning environments [6-8]. Fourth, systems have begun to act rather than only respond. Agentic models that retrieve information, draft documentation, and execute multistep tasks move AI from a source of text toward an active participant in clinical and educational workflows, sharpening questions of oversight and accountability.

This viewpoint proposes that “Generative AI in Medical Education 2.0” should be framed as the transition from advent to entrustment. Entrustment is familiar to medical education through competency-based education and entrustable professional activities (EPAs), where responsibility is granted progressively based on ability, context, risk, and supervision [9-11]. A similar logic can help educators decide how to use AI. The relevant question is whether a defined educational function can be assigned to an AI system in a defined context with appropriate oversight.

This translation has already begun. Gin et al [12] proposed repurposing entrustment to appraise the trustworthiness of AI tools across the characteristics of ability, integrity, and benevolence. A parallel reappraisal of the Association of American Medical Colleges’ core EPAs has proposed emerging activities suited to an AI-rich environment [13]. We build on this work but shift the unit of analysis from the trustworthiness of a tool to the delegation of a defined educational function. Our contribution is operational: a graduated model that pairs each level of delegation with explicit boundaries on stakes, supervision, assessment, and governance.

## Why Entrustment Fits the AI Moment

Medical education is structured around graduated responsibility. Learners begin by observing; progress to performing tasks under direct supervision; and then advance to indirect supervision and, ultimately, independent practice. This framework recognizes that competence is contextual: a student may be trusted to take a history in one clinical situation and require close supervision in another. The same logic should apply to AI systems. A tool may be appropriate for generating alternative explanations of a concept and inappropriate for making a progression decision about a learner. A chatbot may be useful for low-stakes history-taking practice and unsafe as the sole source of feedback on professional communication. A model may pass examination questions and still fail when confronted with local protocols, ambiguous patient narratives, incomplete information, or hidden bias. This graduated logic mirrors established learning theories: cognitive apprenticeship and scaffolding describe how expert support is calibrated to a learner’s current ability, the gradual release of responsibility describes how instructors progressively transfer cognitive work from teacher to learner, and distributed cognition and epistemic agency describe how thinking can be distributed across humans and tools without necessarily diminishing the learner’s own agency. Entrustment in the AI era can therefore be understood not only as a governance mechanism but also as an application of these established learning theories to a new class of cognitive tools.

The analogy between students and AI tools has an important limitation that must be recognized. Entrusting a trainee is developmental: trust grows because the learner learns from supervision and remains morally accountable for outcomes. An AI system does neither. It does not learn from a supervisor’s correction, carries no accountability, and may regress without warning when a model is updated. Two distinct judgments therefore sit behind any AI-enabled activity: entrustment of the learner who uses AI, which remains a judgment about a developing professional, and entrustment of the AI to perform a function, which is a judgment about a tool whose behavior is contingent and must be reverified over time. The levels mentioned below describe the second judgment; the first remains governed by existing competency frameworks.

An entrustment lens also avoids 2 common errors. The first is technological determinism, a term used here to describe the assumption that a technology’s capabilities or momentum make adoption inevitable. The second is blanket restriction, the assumption that uncertainty justifies institutional paralysis. Medical education needs structured adoption with explicit boundaries. We therefore propose 6 levels of AI entrustment in medical education. These levels are ordered by the consequence of an unsupervised error rather than by technical sophistication. A function sits higher when an unreviewed AI error would more directly damage a learner’s progression, a fair assessment, or a patient. Figure 1 summarizes these 6 levels and their defining features.

**Figure 1:**
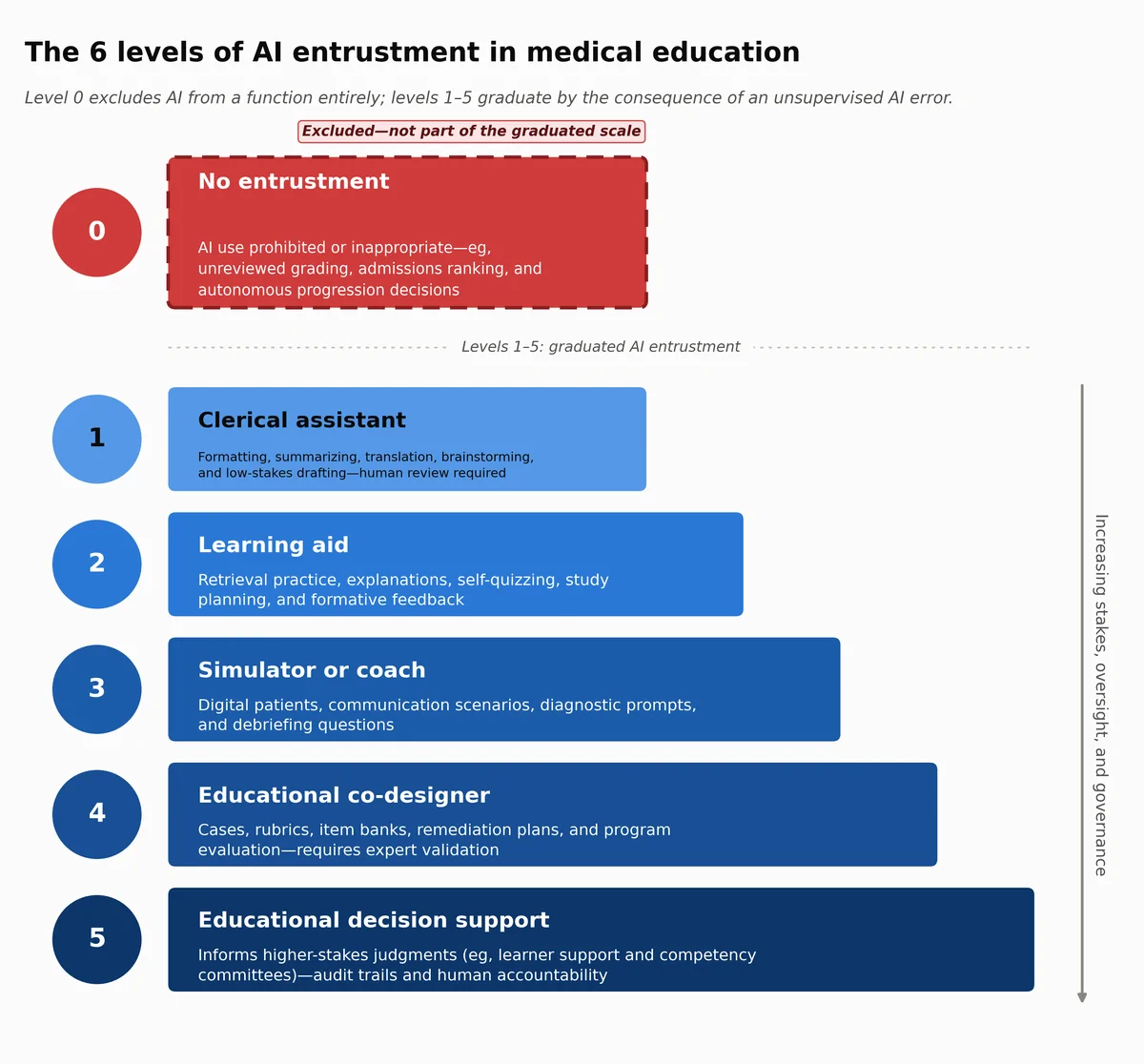
The 6 levels of AI entrustment.

Level 0 is no entrustment. Level 0 is an exclusion category that sits outside the graduated sequence. It identifies functions for which AI entrustment is currently inappropriate, including autonomous or unreviewed decisions about admissions, grading, professionalism, or progression. Levels 1 to 5 describe permitted forms of AI participation with increasing consequence and governance requirements. Educational stakes, AI autonomy, and data sensitivity determine the oversight required for each use.

Level 1 is AI as a clerical assistant. AI helps with formatting, summarizing, translation, brainstorming, and drafting low-stakes educational material. Human review is required, and sensitive data should be excluded unless the tool is institutionally approved.

Level 2 is AI as a learning aid. AI supports retrieval practice, explanations, self-quizzing, study planning, and formative feedback. Learners are taught to verify outputs and document meaningful AI assistance.

Level 3 is AI as a simulator or coach. AI generates digital patients, communication scenarios, diagnostic prompts, or debriefing questions. Faculty define the scenario, review outputs, and monitor whether the tool supports reasoning and communication.

Level 4 is AI as an educational co-designer. AI helps educators build cases, rubrics, item banks, remediation plans, clerkship materials, and program evaluation summaries. Outputs require expert validation, version control, bias review, and documentation.

Level 5 is AI as an educational decision support. AI informs higher-stakes judgments, such as identifying learners who may need support or summarizing multiple data sources for a competency committee. This level requires local validation, transparency, audit trails, human accountability, and appeal mechanisms. Autonomous educational decision-making should remain outside routine use at present.

Figure 1 presents the 6 levels of AI entrustment described above, ordered by the consequence of an unsupervised AI error rather than by technical sophistication. The figure can help curriculum leaders and institutional committees quickly locate a proposed use case and identify the oversight it requires. Level 0 is an exclusion category.

## Use Cases by Stakes and Maturity

The first wave of literature cataloged many possible uses of GenAI. The second wave should distinguish between use cases that are ready for supervised implementation, promising yet evidence-limited, and governance-intensive. Ready uses include generating practice questions, creating alternative explanations, simplifying patient education materials, translating educational content, drafting case stems, suggesting feedback language, and helping faculty overcome blank-page barriers. These functions are usually low stakes, easy to review, and aligned with existing educator judgment. These distinctions also interact with the stage of training. In the nonclinical years, most ready and promising uses are low stakes by nature because errors are easily checked against a still-developing knowledge base; in the clinical years, the same categories of tools intersect directly with patient care, documentation, and time-pressured decision-making, which raises the practical stakes of the supervision described below, particularly for ambient documentation and clinically embedded decision support.

Promising uses include AI standardized patients, adaptive tutoring, individualized remediation, simulation debriefing, communication coaching, and clinical reasoning scaffolds. These applications could transform learning because they create repeated opportunities for practice, feedback, and reflection. Early studies suggest that GenAI can support engagement through multimodal narratives and can be explored for medical interview training, although many studies remain small and context specific [14,15]. These uses deserve careful expansion through multi-institutional trials and direct comparison with existing educational approaches. These promising uses are also well suited to remote and geographically distributed learning, where AI standardized patients, simulation debriefing, and adaptive tutoring can extend a form of faculty presence to learners at affiliated clinical sites or in distance-based coursework, provided the same expectations for supervision, local validation, and equitable access described throughout this viewpoint are maintained.

Governance-intensive uses include summative grading, automated assessment of professionalism, admissions screening, progression recommendations, and any application involving sensitive student or patient data. They now also include agentic tools that take actions across systems and ambient documentation tools that draft clinical notes during real encounters, where the educational question is not only whether the output is accurate but whether the learner still performs the underlying cognitive work. These uses combine high educational stakes with risks of opacity, bias, data leakage, and contested accountability, meaning uncertainty about who owns and is responsible for an AI-mediated educational product or decision when it is inaccurate, harmful, inequitable, or disputed. Institutions should treat them as requiring formal review, similar to educational research protocols or clinical AI implementations.

## From Demonstrations to Educational Workflows

A persistent problem in the AI literature is the gap between demonstration and implementation. Showing that a model can answer a question, draft a case, or imitate a patient is useful; however, educational value depends on workflow. A medical school adopting AI for simulation should specify who writes the scenario, who validates the clinical content, how learner data are stored, how bias is monitored, how students are oriented, and what faculty do when AI produces unsafe or misleading responses. A clerkship using AI for feedback should decide whether the feedback is private practice feedback, formative faculty-reviewed feedback, or evidence that enters the learner record. These distinctions matter because the same technical tool can have very different educational and ethical meanings depending on where it sits in the program.

Implementation should begin with the educational problem. Educators should ask the following: What learning gap are we addressing? What human activity is AI meant to augment? What evidence would count as improvement? What harms are plausible? and Who remains accountable? Health professions education has already emphasized the need for educators to understand AI as part of their professional role [16]. The next step is to translate that understanding into local operating procedures. Each AI-enabled educational activity should have a named owner, a defined learner population, a statement of permitted data, a review process for generated content, and a plan for evaluating outcomes. Tool version, date of use, and local modifications should be documented because model behavior can change over time.

A practical implementation sequence would include 5 steps. First, classify the use case by stakes, autonomy, and data sensitivity. Second, choose an approved tool and specify privacy boundaries. Third, conduct local validation with faculty and learners before full deployment. Fourth, monitor outputs, learner experience, and equity effects during implementation, and, for tools that take actions, log those actions for review. Fifth, revise or retire the activity when evidence, model behavior, or curricular priorities change. This sequence would make GenAI adoption more consistent with the quality improvement culture already familiar to medical schools.

Programs should also treat GenAI as an evolving educational intervention rather than a fixed resource. A printed handout can remain stable for years, but model behavior, vendor policies, training data, and user interfaces may change with little warning. Local validation therefore cannot be a one-time event. Schools should document the date, model, settings, prompt templates, review process, and intended use for each AI-enabled activity. This documentation would make it easier to reproduce educational materials; investigate errors; and explain decisions to learners, faculty, accreditors, and patients.

## Assessment After GenAI

Assessment is the most urgent domain for redesign. Early debate often framed GenAI as a plagiarism problem. That framing is too narrow. GenAI challenges assessment validity because it changes what written products represent. A polished essay may reflect a learner’s understanding, the learner’s ability to frame the task through prompts, the model’s capabilities, and the learner’s decisions about verification and revision. Prompting can itself be intellectual work when it involves problem framing, strategic questioning, iterative critique, and synthesis. Ownership of the final product remains with the learner. The learner is accountable for accuracy, fairness, citation practices, and clinical appropriateness regardless of the extent of AI assistance. Detection tools cannot carry the full burden of academic integrity. Assessment systems should specify when AI use is prohibited, permitted, expected, or required.

AI-independent assessment is appropriate when the educational goal is to verify unaided competence. Examples include observed clinical encounters, oral examinations, in-person reasoning exercises, procedural skills assessments, closed-resource tests, and direct workplace-based assessments. These formats remain essential because physicians must still act under uncertainty, communicate with patients, and make accountable judgments.

AI-assisted assessment is appropriate when AI use resembles real professional practice. Learners may use AI to draft, revise, translate, or structure work, provided they disclose the tool, describe the nature of assistance, and submit evidence of their reasoning process. Such assessments should evaluate verification, critique, and revision rather than final prose alone.

AI-integrated assessment is appropriate when the learning objective is safe and compatible with AI use. Learners might compare AI-generated differential diagnoses, identify hallucinations, check outputs against guidelines, rewrite biased patient education materials, explain how AI altered their thinking, or counsel a simulated patient who arrives having already consulted an AI tool. The Association for Medical Education in Europe Guide on AI in health professions education assessment emphasizes that AI affects assessment types, competencies, ethics, faculty development, and acknowledgment practices [8]. A pilot study also showed that AI performance varies substantially by assessment type, performing strongly in rule-based and reflective tasks while struggling with technical accuracy and contextual application in some health information management tasks [17]. This reinforces a practical conclusion that the assessment design should make reasoning visible.

## Curriculum and Faculty Development

AI literacy is becoming a core professional competency. A curriculum focused only on prompt technique would be insufficient. Learners need conceptual, ethical, epistemic, and practical competence. They should understand how LLMs generate outputs, why hallucinations occur, how bias enters training and deployment, how privacy can be compromised, and why fluent language can obscure weak reasoning. They should also learn how to verify outputs, cite or acknowledge AI assistance, recognize automation bias, and preserve accountability. They should also learn to work with patients who arrive already informed (or misinformed) by AI tools, correcting errors without dismissing the patient’s engagement. Managing the AI-informed patient is plausibly an emerging entrustable activity in its own right.

Medical students have expressed interest in AI, while also reporting variable preparedness and concern about ethics, curriculum gaps, and future clinical roles [18]. Reviews of AI curricula emphasize that medical education needs structured frameworks, progressive sequencing, and integration with clinical contexts rather than isolated technical electives [19,20]. Practical AI literacy courses for early medical students and calls for AI ethics training provide starting points, but curriculum design should extend across undergraduate, graduate, and continuing medical education [21,22]. Curricula will also need validated measures. AI literacy and susceptibility to automation bias are currently assessed inconsistently, and the field lacks agreed-upon instruments for certifying that learners can use AI safely. Identifying and closing this measurement gap is itself a curricular priority.

Faculty development is equally important. Educators require enough understanding to supervise AI use responsibly. Faculty need training in tool selection, prompt design, output evaluation, bias recognition, privacy rules, assessment redesign, and learner coaching. They also need time and institutional support. Without faculty development, AI integration may default to uneven local experimentation, with wide variation in quality and access.

## Cognitive Autonomy and Professional Identity

A mature sequel to the first wave of GenAI literature must address cognitive autonomy. Medical education is partly a process of productive struggle. Learners develop clinical judgment by generating hypotheses; being wrong; receiving feedback; revising mental models; and integrating biomedical, psychosocial, and contextual information. If AI systems prematurely remove that struggle, learners may become efficient producers of polished answers while developing weaker habits of inquiry.

The risk extends beyond student cheating to cognitive outsourcing. A learner who repeatedly asks AI to generate differential diagnoses may gradually lose the habit of constructing them independently. The clearest current example is ambient clinical documentation. Students increasingly rotate through clinics where an AI scribe drafts the note in real time. The convenience is real, but synthesizing a patient’s story into an assessment and plan is precisely the reasoning work that builds clinical judgment. A learner who never drafts the note may never practice the synthesis. A faculty member who repeatedly asks AI to draft feedback may become less attentive to the learner’s actual performance. A program that relies on AI-generated dashboards may confuse data aggregation with educational judgment. Recent work on AI dependency among educators found perceived risks to skills, pedagogy, motivation, ethics, collaboration, and creativity, highlighting the need to study overreliance at the faculty level as well as the learner level [23]. Reviews of GenAI and professionalism similarly emphasize that technological fluency must be paired with empathy, integrity, accountability, and humanistic practice [24]. The cognitive work that should remain the learner’s own is therefore specific rather than diffuse: generating an initial differential from first principles, synthesizing an undifferentiated encounter into an assessment and plan, weighing conflicting or incomplete evidence, and forming a working hypothesis under uncertainty before consulting any external aid. An AI-generated differential or note draft is a representation the learner can review and critique; it is not evidence that the learner has performed the reasoning that representation implies, and assessment should be designed to tell the two apart.

At the same time, AI may strengthen cognition when used deliberately. It can serve as a debate partner, generate counterarguments, expose learners to alternative explanations, and support diagnostic reflection. Studies of diagnostic reasoning suggest that AI tools may influence clinician reasoning and that prompting models to display reasoning can make outputs more interpretable [25,26]. The educational goal should be hybrid intelligence: learners who can think independently, use AI strategically, recognize when AI is misleading, and remain accountable for final judgments.

## Governance and Equity

Institutional governance should move beyond academic honesty statements. A recent audit of AI-related documents across US medical schools found that many documents emphasized academic integrity and plagiarism, while audit mechanisms, technical infrastructure, and long-term planning were less commonly addressed [27]. Medical schools should develop policies that define approved tools, prohibited uses, disclosure expectations, privacy safeguards, data retention rules, procurement standards, bias monitoring, assessment categories, incident reporting, and periodic review. Textbox 1 summarizes minimal institutional commitments for generative AI in medical education. These commitments should be written in plain language, revisited regularly, and aligned across undergraduate medical education, graduate medical education, faculty development, and institutional compliance. A policy that focuses only on plagiarism leaves too many educational and safety questions unanswered.

**Minimum institutional commitments for generative AI in medical education.**

Six operational questions every medical school should be able to answer before scaling AI use:

1. Which tools are approved for learners and faculty?
2. What data may be entered, and what data are prohibited?
3. When must AI assistance be disclosed?
4. Which assessments are AI-independent, AI-assisted, or AI-integrated?
5. Who reviews AI-generated educational content before learners see it?
6. How will the school monitor errors, bias, learner overreliance, access gaps, and model changes?

Approving a tool for institutional use should weigh, at minimum, the vendor’s data privacy and retention practices, evidence of accuracy and bias testing relevant to the intended educational use, transparency about model versioning and training data, compatibility with institutional authentication and data security requirements, cost and licensing implications for equitable access, and a designated process for periodic rereview as the tool or its underlying model changes.

Governance should also address equity. The earlier “digital divide” now includes an “AI divide.” Students and schools vary in access to paid models, high-quality institutional tools, secure platforms, faculty expertise, broadband, language support, and protected time for training. Learners in low-resource settings may face restricted access to the most capable systems while also being more exposed to unvetted free tools. Equity also includes disability access, language performance, cultural representation in cases, and fair treatment of learners whose writing style is flagged as suspicious. An institution that permits AI use without ensuring equitable access may inadvertently advantage learners with greater resources.

Policy language should be role-specific. Learners need clear expectations for disclosure and verification. Faculty need guidance for creating materials, providing feedback, and documenting AI assistance. Administrators need procurement criteria, data protection rules, and processes for reviewing vendors. Competency committees need boundaries around how AI-generated summaries may be used. Role-specific policy reduces ambiguity and makes responsible AI use easier to teach, supervise, and audit. Successful implementation also depends on stakeholders beyond faculty and learners. Information technology and data security personnel are needed to vet vendor data-handling practices, configure institutional access controls, and monitor for data leakage, while instructional designers and clinical informaticists help translate approved tools into workflow-embedded activities; governance structures should include these roles formally rather than treating AI oversight as an academic affairs function alone.

## Research Agenda

The next research agenda should prioritize educational outcomes. The literature already contains many studies asking whether AI can answer examination questions. Medical education now needs studies asking whether AI improves durable learning, diagnostic calibration, communication, feedback quality, clinical performance, and patient outcomes. A systematic review of GenAI in health professional education found that student use aligns with several learning activities, while evidence remains limited by short duration, small samples, and underexplored collaborative learning [28]. The field needs longitudinal, multicenter, theory-informed studies. The field is also moving past the point where novelty alone justifies attention; the priority now is whether documented learning gains persist and transfer to practice.

Key research questions include the following: Which AI tutoring designs improve retention and transfer? How does AI feedback compare with faculty feedback across clinical skills? Does AI-assisted simulation improve real patient communication? Which forms of AI disclosure support integrity without stigmatizing appropriate use? What learner groups are helped or harmed? How do AI tools affect workload, burnout, creativity, and teaching quality among faculty? What governance structures reduce risk while supporting innovation? How should institutions evaluate model updates that alter performance over time? What level of autonomy can agentic AI safely assume in educational tasks, and how should action-taking tools be evaluated differently from advisory ones? and Which validated instruments reliably measure AI literacy, automation bias, and appropriate reliance, and how do these correlate with clinical performance?

## The AI Entrustment Matrix

Table 1 presents a conceptual matrix with 2 axes: educational stakes, from low to high, and AI autonomy, from assistant to decision support. The matrix can help curriculum leaders decide when routine disclosure is sufficient and when formal governance is required.

**Table 1:** A conceptual matrix for evaluating AI entrustment in the educational environment.

	Low educational stakes	High educational stakes
Low AI autonomy	Accept with disclosure and ordinary faculty review. Examples: brainstorming, formatting, translation, and practice questions	Use with validation, documentation, and accountable human review. Examples: rubric drafting, case design, and feedback support
High AI autonomy	Permit for practice environments with monitoring for overreliance. Examples: AI standardized patients and adaptive tutoring	Restrict or formally govern. Examples: progression recommendations, automated grading, and admissions screening

## Conclusion: From Adoption to Accountability

GenAI has already entered medical education. The task now is to shape its role deliberately. The first phase established that these tools could generate plausible educational content, simulate conversations, support writing, and assist learning. The second phase must determine how, when, and under what supervision they should be used. Entrustment offers a language that medical educators already understand: responsibility should be granted progressively, contextually, and with evidence.

Medical schools should develop AI policies that are practical, transparent, and revisable; assessments that make reasoning visible; curricula that teach AI literacy as part of professionalism; and research programs that evaluate learning rather than novelty alone. GenAI should be judged by its effects on learners, educators, institutions, and ultimately patients. The goal is accountable integration: using AI to expand educational possibility while preserving the human judgment at the center of medicine. Although this framework is presented for medical education, the underlying questions, namely, which cognitive functions to delegate, to what degree, and with what oversight, are not unique to medicine. Educators across science, technology, engineering, and mathematics (STEM) disciplines and other professional fields built around graduated responsibility, including engineering, nursing, and teacher education, face comparable decisions about entrusting AI with defined instructional functions and may find this entrustment framework a useful starting point for their own context.

## Abbreviations

EPA: entrustable professional activity
GenAI: generative AI
LLM: large language model
STEM: science, technology, engineering, and mathematics
